# Optimal discharge planning for esophagectomy patients with enhanced recovery after surgery: Recommendations

**DOI:** 10.3389/fsurg.2023.1112675

**Published:** 2023-01-30

**Authors:** Kunzhi Li, Kangning Wang, Xing Wei, Xuefeng Leng, Qiang Fang

**Affiliations:** Division of Thoracic Surgery, Sichuan Cancer Hospital & Institute, Sichuan Cancer Center, School of Medicine, University of Electronic Science and Technology of China, Chengdu, China

**Keywords:** esophageal cancer, esophagectomy, enhanced recovery after surgery, postoperative length of stay, complications, comorbidities

## Abstract

**Background:**

Studies have suggested that the postoperative length of stay (PLOS) of esophagectomy patients under the enhanced recovery after surgery (ERAS) pathway should be >10 days as against the previously recommended 7 days. We investigated the distribution and influencing factors of PLOS in the ERAS pathway in order to recommend an optimal planned discharge time.

**Methods:**

This was a single-center retrospective study of 449 patients with thoracic esophageal carcinoma who underwent esophagectomy and perioperative ERAS between January 2013 and April 2021. We established a database to prospectively document the causes of delayed discharge.

**Results:**

The mean and median PLOS were 10.2 days and 8.0 days (range: 5–97), respectively. Patients were divided into four groups: group A (PLOS ≤ 7 days), 179 patients (39.9%); group B (8 ≤ PLOS ≤ 10 days), 152 (33.9%); group C (11 ≤ PLOS ≤ 14 days), 68 (15.1%); group D (PLOS > 14 days), 50 patients (11.1%). The main cause of prolonged PLOS in group B was minor complications (prolonged chest drainage, pulmonary infection, recurrent laryngeal nerve injury). Severely prolonged PLOS in groups C and D were due to major complications and comorbidities. On multivariable logistic regression analysis, open surgery, surgical duration >240 min, age >64 years, surgical complication grade >2, and critical comorbidities were identified as risk factors for delayed discharge.

**Conclusions:**

The optimal planned discharge time for patients undergoing esophagectomy with ERAS should be 7–10 days with a 4-day discharge observation window. Patients at risk of delayed discharge should be managed adopting PLOS prediction.

## Introduction

Esophageal cancer is the seventh most commonly diagnosed cancer in the world and the sixth most common cause of cancer-associated mortality (1). More than 500,000 people die of this disease each year (1, 2). Currently, surgery is the cornerstone of multidisciplinary treatment for resectable esophageal cancer. The concept of enhanced recovery after surgery (ERAS) was first introduced by Professor Henrik Kehlet in Denmark in 1997. Over the subsequent years, the ERAS pathway has rapidly evolved and has been applied to all fields of surgery. ERAS model has been shown to significantly reduce the length of hospital stay without increasing complications (3–5). Most studies have demonstrated the safety, feasibility, and effectiveness of ERAS in patients with esophageal cancer (6–8); however, there is no clear consensus whether this approach can shorten the postoperative length of stay (PLOS). In the traditional perioperative pathway for esophageal cancer surgery, discharge time is usually determined according to the patient's postoperative status, and there is no emphasis on establishing a uniform, planned PLOS. In contrast, in the esophagectomy ERAS pathway, the currently accepted PLOS ends on postoperative day 7 (9). However, most studies have reported a mean or median PLOS of >10 days (6, 10, 11). As esophagectomy is a complex major surgery with many complications, a proportion of patients do not successfully complete the standard full perioperative protocol in the ERAS model of esophagectomy, resulting in delayed discharge. In the international esophageal cancer dataset (Esodata) study, in which our institution is a participating center, the overall rate of perioperative complications was as high as 60%, and only approximately 40% of patients experienced no complications (12). Esophagectomy ERAS does not significantly increase complications compared to conventional surgery, but the distribution of PLOS is not well characterized. Further research to develop a standardized PLOS for these patients is a key imperative. In addition, neoadjuvant therapy has gradually been introduced as a standard treatment option in esophageal cancer surgery (13, 14); however, its impact on the PLOS of ERAS is yet to be determined. In this study, we investigated the distribution and influencing factors of PLOS in patients undergoing ERAS esophagectomy from the beginning of the implementation of ERAS pathway at our institution in January 2013. Our findings may help establish a reasonable target PLOS for this population.

## Methods

### Data collection

This was a single-center retrospective cohort study of 449 patients with thoracic esophageal cancer who were treated at the Department of Thoracic Surgery between January 01, 2013 and April 30, 2021. All patients underwent surgery, and perioperative ERAS measurements were performed by the same team. The study was conducted according to the principles enshrined in the Declaration of Helsinki and the study protocol was approved by the Ethics Committee (EC) for Medical Research and New Medical Technology of Sichuan Cancer Hospital (SCCHEC-02-2022-042). Written informed consent of patients was waived by the EC. Data pertaining to clinical status, tumor characteristics, neoadjuvant therapy, surgical approach, surgical duration, intraoperative blood loss, anastomotic approach, anesthesia, PLOS, and perioperative complications (severity assessed using the Clavien-Dindo grading scale) were recorded. Causes of delayed discharge were monitored prospectively.

### Diagnosis, treatment, and the standards for discharge from hospital

Clinical staging was based on preoperative contrast-enhanced cervicothoracic and abdominal CT, endoscopic ultrasound, fibreoptic bron choscopy, neck and abdominal ultrasound, and bone scan, with additional contrast-enhanced head MRI, and PET-CT scans to rule out distant metastases, if necessary. Tumor pathology was staged according to the 8th edition of the International Union Against Cancer (UICC). Neoadjuvant chemoradiotherapy, chemotherapy, or immunochemotherapy was administered according to the National Comprehensive Cancer Network guidelines, the Chinese Society of Clinical Oncology guidelines, registered clinical trials, and the patients’ wishes. Patients were treated with McKeown esophagectomy and three-field lymph node dissection if the tumor was located in the thoracic segment; the Ivor-Lewis or transhiatal surgical approach was used for tumors located at the esophagogastric junction. Clinical protocols of patients undergoing esophagectomy are shown in Table 1. The criteria for removal of the thoracic drainage tube were: no air leak, pleural drainage fluid <100 ml/day, and non-cloudy pleural drainage fluid. Patients were discharged when they qualified the following criteria: no need for intravenous rehydration, transoral semi-liquid diet with soft food that can meet physiological needs, normal body temperature, white blood cell count and neutrophil count within the normal range, wound healing grade A, ability to move freely when getting out of bed, no other serious complications, and willingness to be discharged.

**Table 1:** Standardized ERAS protocol for perioperative.

Postoperative day	Daily Goals and Scheduled ERAS Item (s)
Admission	Comprehensive screening and assessment
Preoperative preparation	Complete auxiliary examinations; adjust blood sugar, blood pressure, nutritional status
−1	expectoration and breathing training; ERAS education; thrombosis prevention;
Before anesthesia	Oral 50 g glucose water 4 h before anesthesia
Operation	Surgical approaches: Minimally invasive esophagectomy (MIE) or open thoracotomy preserving the serratus anterior
	Surgical methods: pyloroplasty, stapler anastomosis
	No drainage tube placed near the anastomosis
	No gastric tube
	No jejunal feeding tube
0	Relieving pain with intravenous analgesic pump; rehydration; if there is no hoarseness, drink 100–200 ml of warm water 6–8 h after surgery
1	5 min sport in bed
	Oral intake of water or clear liquid, a total of 500 ml
	Parenteral Nutrition; Fluid Control
	Radiology tests, blood tests
2	Mobilization; remove urinary catheter; oral fluid or semi-liquid 500–1,000 ml
3	Fluid control; chest tube removal assessment; oral semi-fluid 500–1,000 ml
	Radiology tests, blood tests
4–6	Eating soft food; appropriate amount of fluid replacement or discontinuation of fluid replacement as appropriate
	Chest tube removal assessment
7	Radiology tests, blood tests
	Discharge assessment

### Statistical analysis

Data analyses were performed using SPSS 24.0 software. Normally distributed continuous variables were expressed as mean ± standard deviation and between-group differences assessed using independent samples *t* test. Non-normally distributed continuous variables were expressed as median (range) and between-group differences assessed using the Mann-Whitney *U* test. Categorical variables were expressed as frequency (percentage) and between-group differences assessed using the Chi-squared test or Fisher's exact probability method. All statistically significant perioperative variables were included in a multifactorial analysis model using binary logistic stepwise regression analysis. Variables that were not statistically different were phased out until the model was explained by the lowest number of variables. *P* values < 0.05 were considered indicative of statistical significance.

## Results

### Descriptive characteristics

A total of 449 patients (358 men, 91 women; mean age: 63.1 ± 8.7 years) who were selected for the perioperative ERAS pathway were included in this study. The baseline characteristics are summarized in Table 2. Neoadjuvant therapy was used in 99 (22.0%) patients, and 82.8% of these patients had received neoadjuvant therapy within the past 4 years. Neoadjuvant chemoradiotherapy was administered to 75 patients, whereas 5 patients received neoadjuvant immunochemotherapy, 18 patients received neoadjuvant chemotherapy, and 1 patient received neoadjuvant radiotherapy. Esophagectomy was performed 4–8 weeks after neoadjuvant therapy.

**Table 2:** Patient characteristics and preoperative data.

Variables	*N* = 449 (%)
Age (mean, range)	*63.1* (*36–84)*
**Sex**	
Male	358 (79.7)
Female	91 (20.3)
**Location**	
Upper chest	94 (20.9)
Middle chest	250 (55.7)
Lower chest	105 (23.4)
**T**	
0	36 (8.0)
1	57 (12.7)
2	80 (17.8)
3	190(42.3)
4	86 (19.2)
**N**	
0	234 (52.1)
1	117 (26.1)
2	62 (13.8)
3	36 (8.0)
**Surgical modalities**	
McKeown	276 (61.4)
Ivor-Lewis	166 (37.0)
Transhiatal	7 (1.6)
**Anastomosis**	
End-to-side anastomosis	255 (56.8)
Side-to-side anastomosis	194 (43.2)
**Neoadjuvant therapy**	
No	350 (78.0)
Yes	99 (22.0)

### Distribution of PLOS

**Figure 1:**
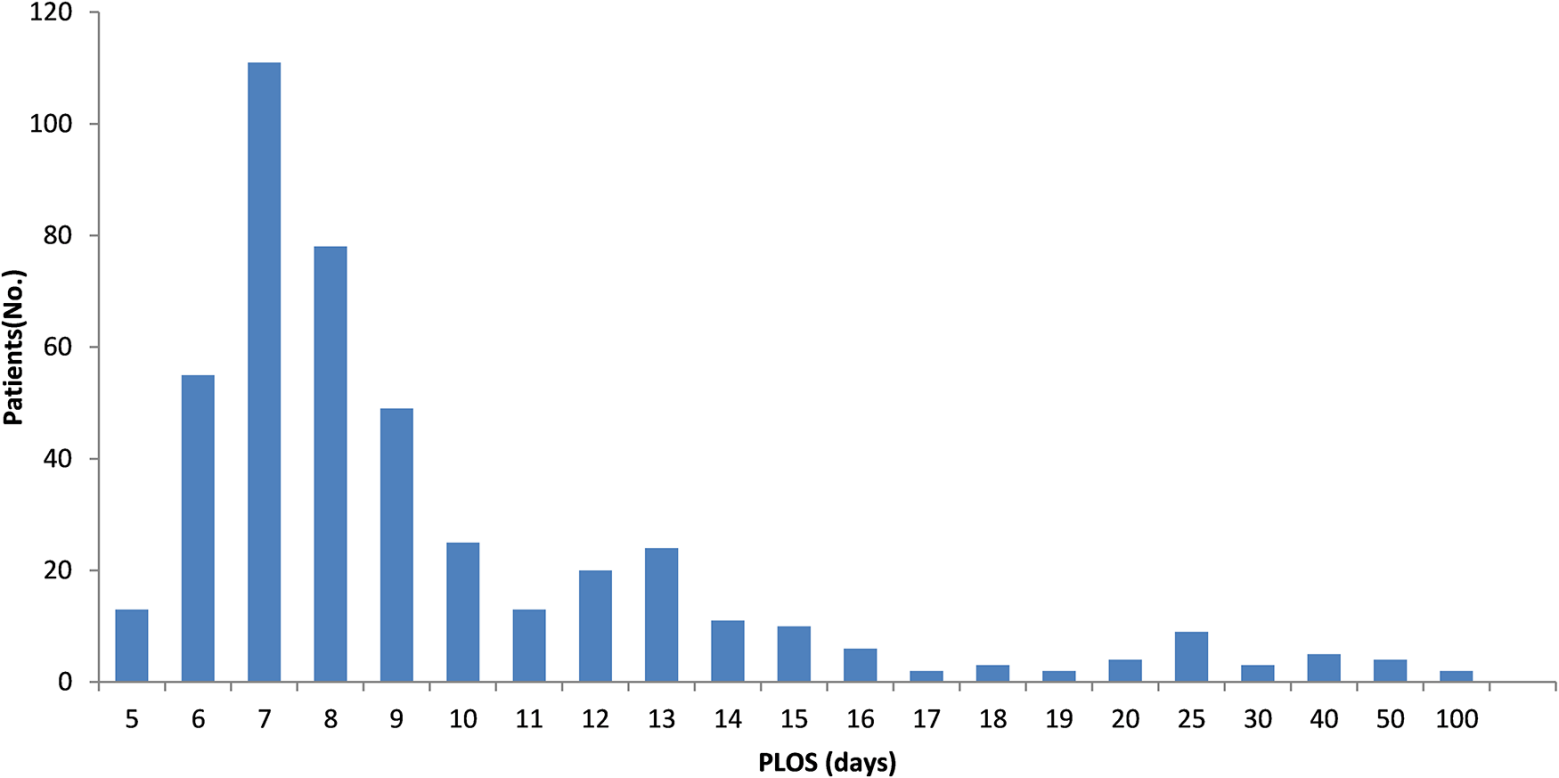
Postoperative hospital length of stay, patient distribution. PLOS, postoperative length of stay; Patients (No.), the number of patients;.

The distribution of the PLOS in our cohort is shown in Figure 1. The mean and median PLOS were 10.2 and 8.0 (range 5–97) days, respectively. Patients were divided into four groups according to their PLOS: group A (PLOS ≤ 7 days, *n* = 179, 39.9%); group B (8 ≤ PLOS ≤ 10 days, *n* = 152, 33.9%); group C (11 ≤ PLOS ≤ 14 days, *n* = 68, 15.1%); and group D (PLOS > 14 days, n = 50, 11.1%). The PLOS data had a skewed distribution. The PLOS of 73.8% patients was within the range of 3 days from the standard PLOS plan (5 ≤ PLOS ≤ 10 days, groups A and B).

### Reasons for delayed discharge

The most important reason for delayed discharge was postoperative complications (Table 3). Patients in group B had mild complications, the most common of which were prolonged chest drainage time, pulmonary infection, recurrent laryngeal nerve injury, pulmonary air leak, and electrolyte abnormalities. Patients in group C had moderate complications, the most common of which were prolonged chest drainage, recurrent laryngeal nerve injury, poor incision healing, pulmonary infection, and gastrointestinal disorders. Pulmonary impairment was the most significant comorbidity leading to delayed discharge. More serious complications occurred in group D, most notably anastomotic fistulas and pulmonary infection. Prolonged chest drainage and pulmonary air leakage were important causes of complications, which may be closely related to the poor lung function experienced by those with preoperative comorbidity, leading to significant pulmonary and liver function impairment.

**Table 3:** Reasons for delayed discharge.

	Group B (8–10)	Group C (10–14)	Group D (>14)	Total (%) *n* = 270
Complications	140(92.18%)	57 (83.8%)	46 (92.0%)	243 (90.0%)
Anastomotic leakage	3 (2.0%)	1 (1.5%)	20 (40.0%)	
Pulmonary infection	35 (23.0%)	6 (8.8%)	11 (22.0%)	
Recurrent laryngeal nerve injury	12 (7.9%)	11 (16.2%)	3 (6.0%)	
Prolonged chest drainage	75 (49.3%)	20 (29.4%)	6 (12.0%)	
Pneumothorax	9 (5.9%)	3 (4.4%)	5 (10.0%)	
Poor wound healing	6 (3.9%)	8 (11.8%)	2 (4.0%)	
Chylothorax	4 (2.6%)	3 (4.4%)	2 (4.0%)	
Gastrointestinal disorders	9 (5.9%)	9 (13.2%)	2 (4.0%)	
Arrhythmia	6 (3.9%)	2 (2.9%)	0 (0%)	
Electrolyte disorders	7 (4.6%)	3 (4.4%)	0 (0%)	
Bleeding	2 (1.3%)	2 (2.9%)	0 (0%)	
Comorbidities	15 (9.9)	11 (16.2)	7 (14.0)	33 (12.2%)
Lung function loss	4 (2.6%)	11 (16.2%)	3 (6.0%)	
Kidney function loss	3 (2.0%)	0 (0%)	0 (0%)	
Liver function loss	8 (5.3%)	0 (0%)	4 (8.0%)	
Special surgical modality	1 (0.7%)	2 (2.9%)	1 (2.0%)	4 (1.5%)

### Readmission

Six patients were readmitted within one month of discharge (readmission rate: 1.3%). Two patients were in group A, three in group B, and one in group C. All six patients were readmitted because of complications [anastomotic leakage (four patients), aspiration pneumonia (two patients), and recurrent laryngeal nerve injury (one patient)]. Clinical manifestations and blood indices of these patients were within the normal range, and all readmitted patients were eventually discharged (Table 4).

**Table 4:** General clinical data of patients readmitted within 1 month after discharge.

	1	2	3	4	5	6
WBC (10^9^/L)	5.30	6.68	5.73	4.37	4.56	7.55
CRP (mg/L)	33.21	36.90	30.83	10.86	57.19	31.03
Temperature (°C)	36.0	36.3	36.6	37.3	37.0	36.8
Pulse rate (/min)	79	70	91	100	89	81
PLOS (d)	7	7	8	8	9	12
Reason	Anastomotic leakage	Anastomotic leakage	Aspiration pneumonia	Anastomotic leakage	Anastomotic leakage	Aspiration pneumonia
Readmission time (d)	11	9	15	9	13	18
Length of hospital stay again (d)	11	11	5	14	28	6

WBC, white blood cell; CRP, C reactive protein; PLOS, postoperative length of stay; Reason, reason for readmission.

The readmission time is patient readmission time after operation. The length of hospital stay again is length of hospital stay for patients readmitted.

### Factors influencing PLOS

In this study, there were no significant differences between patients with PLOS ≤10 days and those with PLOS >10 days with respect to sex, tumor location, T stage, N stage, surgical approach, or neoadjuvant therapy. However, there were significant differences between the two groups with respect to age (*P* < 0.001), access modality (*P* = 0.04), American Society of Anesthesiologists (ASA) score (*P* = 0.01), intraoperative bleeding (*P* = 0.01), surgical duration (*P* = 0.04), classification of surgery-related complications (*P* < 0.001), and severe comorbidities (*P* < 0.001) (Table 5). There was no significant difference with respect to the PLOS between the neoadjuvant group (8 days, range, 5–97 days) and the non-neoadjuvant group (8 days, range 5–51 days, *P* = 0.88). PLOS score, operative time, intraoperative bleeding, and age were transformed into categorical variables.

**Table 5:** Univariable analysis of delayed discharge.

Variables	Total (*n* %) 449	PLOS		*χ* ^2^	*P*
		≤10 (331)	>10 (118)		
Age (median)	64 (56–69)	63 (56–68)	67 (61–72)		<0.001^a^
Sex				1.091	0.35^b^
Male	358 (79.7)	260 (78.5)	98 (83.1)		
Female	91 (20.3)	71 (21.5)	20 (16.9)		
Location				0.134	0.95^b^
Upper chest	94 (20.9)	70 (21.1)	24 (20.3)		
Middle chest	250 (55.7)	185 (55.9)	65 (55.1)		
Lower chest	105 (23.4)	76 (23.0)	29 (24.6)		
T				1.436	0.28^b^
T ≤ 3	363 (80.8)	272 (82.2)	91 (77.1)		
T > 3	86 (19.2)	59 (17.8)	27 (22.9)		
N				0.033	1.00^b^
N ≤ 2	413 (92.0)	304 (91.8)	109 (92.4)		
N > 2	36 (8.0)	27 (8.2)	9 (7.6)		
Access modality				4.653	0.04^b^
MIE	378 (84.2)	286 (86.4)	92 (78.0)		
Open	71 (15.8)	45 (13.6)	26 (22.0)		
Surgical approaches				0.464	0.51^b^
McKeown esophagectomy	276 (61.5)	206 (62.2)	70 (59.3)		
Ivor-Lewis esophagectomy	166 (37.0)	119 (36.0)	47 (39.8)		
Transhiatal esophagectomy	7 (1.5)	6 (1.8)	1 (0.9)		
Neoadjuvant therapy				0.069	0.80^b^
NO	350 (78.0)	257 (77.6)	93 (78.8)		
YES	99 (22.0)	74 (22.4)	25 (21.2)		
ASA				7.091	0.01^b^
≤2	422 (94.0)	317 (95.8)	105 (89.0)		
>2	27 (6.0)	14 (4.2)	13 (11.0)		
Intraoperative blood loss	150 (100–200)	150 (100–200)	200 (100–300)		0.01^a^
Surgical duration	240 (213.5–270)	240 (210–270)	250 (215–300)		0.04^a^
Surgical Complications				43.868	<0.001^b^
≤2	429 (95.5)	329 (99.4)	100 (84.7)		
>2	20 (4.5)	2 (0.6)	18 (15.3)		
Comorbidities				21.572	<0.001^b^
NO	401 (89.3)	309 (93.4)	92 (78.0)		
YES	48 (10.7)	22 (6.6)	26 (22.0)		

Due to the small number of cases of transhiatal esophagectomy in the surgical procedure, this group was excluded from univariable analysis.

^a^ Mann-Whitney *U* test.

^b^ χ^2^ test or Fisher's exact probability.

The severity of the complications are evaluated using the Clavien-Dindo grading scale.

Variables that showed significant differences in the univariable analysis were included in the multivariable binary logistic regression models. Multivariable binary logistic regression was performed after excluding patients with intraoperative blood loss of >150 ml (*P* = 0.515, OR = 1.180, 95% CI: 0.717–1.942) and ASA score >2 (*P* = 0.244, OR = 1.717, 95% CI: 0.692–4.261). The Hosmer-Lemeshow test revealed that the model explained 23.9% (Nagelkerke R2) of delayed discharges and correctly predicted 78.2% of discharges, with a sensitivity of 95.5%, specificity of 29.7%, positive predictive value of 79.2%, and a negative predictive value of 70.0%. The results of the multivariable binary logistic regression model are shown in Table 6; surgical duration >240 min [odds ratio (OR) = 1.903, 95% confidence interval (CI): 1.184–3.057], age >64 years (OR = 2.218, 95% CI: 1.376–3.575), surgery-related complication grade >2 (OR = 44.378, 95% CI: 9.719–202.632), and severe comorbidities (OR = 4.183, 95% CI: 2.199–7.985) were identified as significant factors affecting the PLOS.

**Table 6:** Multivariable analysis of delayed discharge.

Variables	B	S.E.	P.	OR	95%CI (OR)	
					Lower	Upper
Open	0.542	0.306	0.077	1.719	0.943	3.135
Surgical duration >240 min	0.643	0.242	0.008	1.903	1.184	3.057
Age > 64	0.797	0.244	0.001	2.218	1.376	3.575
Surgical Complication grade >2	3.793	0.775	<0.001	44.378	9.719	202.632
Comorbidities	1.431	0.328	<0.001	4.183	2.199	7.985
Constant	−2.208	0.246	<0.001	0.110		

B indicates beta coefficient; CI, confidence interval; OR, odds ratio; S.E., standard error.

## Discussion

The goal of the ERAS pathway is to enable patients to recover and approach a healthy physiological state as soon as possible after esophagectomy (15). Several studies have demonstrated the safety and efficacy of ERAS in esophagectomy (4, 8). Compared to the conventional esophagectomy protocol, the main intraoperative measures in the ERAS protocol include minimally-invasive surgery, pyloroplasty, no placement of drains near the anastomosis, no nasogastric tube, and no feeding tube. The main postoperative measures include early initiation of oral diet with parenteral nutrition, early postoperative removal of the nasogastric tube, and restriction of perioperative fluids (9). In the international multicentre Esodata study, the overall rate of surgery-related complications was approximately 60%, regardless of the surgical approach (minimally-invasive, hybrid, or open surgery). The incidence of Clavien-Dindo grade ≥ III b ranged from 15.7%–19.2%, with a mean length of stay of more than 10 days. Severe surgical complications affect the PLOS (16). Under the traditional clinical pathway, perioperative complications and mortality are core evaluation indicators, whereas PLOS is not (4, 17). In contrast, PLOS is the main indicator for evaluating the success of the standard ERAS pathway. Currently, in the ERAS pathway, the planned discharge time is set to 7 days after surgery. However, the timing and content of a standardized pathway based on this concept have not been well described. There are no specific reports on the number of patients who can successfully complete this pathway. The current lack of evidence prevents surgeons and patients from accurately developing individualized preoperative ERAS protocols and postoperative recovery goals. In this study, the mean and median PLOS were 10.2 days and 8.0 days, respectively. Only 39.9% of the patients were discharged within 7 days, and the majority of patients were unable to meet the planned discharge time according to the standard pathway. Approximately 40% of patients reached the planned standard PLOS without any complications, which is consistent with the results of the International Esodata Study. If the planned PLOS were extended by 3 days, 73.8% of patients would have successfully achieved the target PLOS with the ERAS protocol. The current target PLOS should be further optimized to improve its applicability. In our study, 15.1% and 11.1% of the patients were discharged after 11–14 days and 14 days, respectively. We identified the risk factors for increased PLOS, which may help determine the patient suitability for treatment with the standard ERAS protocol. Our results may help inform strategies for development of individualized ERAS clinical protocols for patients.

The readmission rate in our cohort was 1.3%, which is lower than that cited in the International Esodata Study statistical index (19.4%) (12). Five of the six patients readmitted after discharge belonged to groups A (2 patients) and B (3 patients), and these patients were readmitted because of anastomotic leak or aspiration pneumonia. Even when patients are discharged after successfully completing the standard ERAS pathway, there may be potential for undetectable anastomotic leaks. Four patients had a clinically significant anastomotic leak within 4 days of the planned PLOS discharge. However, the diagnosis of an anastomotic leak is time-related and is determined by leak characteristics. Therefore, patients at risk for anastomotic leak in any aspect of the perioperative period should be strictly evaluated for discharge criteria, with a possible extended discharge observation window, or additional testing to identify subtle clues of leakage. Optimal planning of the PLOS in the ERAS pathway for esophagectomy should be set within a window of approximately 4 days to provide a sufficient observation period for patients at potential risk. This would help maximize the timely detection of leak.

The database on which this study is based was primarily developed to address causes of impact-planned discharges. The database actively records impact events and causes. This is critical for an accurate analysis of the reasons for delayed discharge. Continuous analysis of these reasons can improve the quality of the ERAS protocol and provide a basis for developing the optimal PLOS plan for each patient. In this study, 33.9% of patients were discharged within 8–10 days; whether this group of patients could achieve the standard PLOS pathway (PLOS ≤ 7 days) warrants further analysis. In group B, the reasons for delayed discharge included prolonged chest drainage, pulmonary infection, and minor complications, such as recurrent laryngeal nerve injury, electrolyte disturbance, or pneumothorax. Esophagectomy and lymph node dissection result in large intrathoracic wounds that impede lymphatic return and produce more exudate (18, 19). Postoperative tissue repair and inflammation also increase capillary permeability inducing more drainage (20). Many factors contribute to the prolonged duration of chest drainage after esophagectomy. These factors vary among individual patients and are typically uncontrollable in advance despite being the main causes of prolonged chest drainage time. Pulmonary infection in post-esophagectomy patients is closely associated with advanced age, smoking history, underlying lung disease, and duration of surgery (21). The high incidence of pulmonary infection and individual differences make it difficult for patients in group B to fully meet the planned PLOS discharge criteria, even if they have successfully undergone the surgery and ERAS protocol. Patients tend to recover better if a discharge observation window is appropriately provided. This meets the requirements of ERAS and should be included in the PLOS plan.

In group C, more than 10% of patients had delayed discharge due to severely prolonged drainage, poor pulmonary function, recurrent laryngeal nerve injury, gastrointestinal disturbances, or poor wound healing. More than 5% had delayed discharge due to pulmonary infection. Patients in groups B and C had similar complications; however, complications in group C were more severe, and the patient's underlying condition tended to be worse. In patients with risk factors undergoing esophagectomy, it is particularly important to selectively dissect the lymph nodes surrounding the recurrent laryngeal nerve in order to avoid damage to it (22). This is a key surgical technique to avoid thermal and traction injury to the recurrent laryngeal nerve. The main reasons for delayed discharge in group D were severe anastomotic leak, pulmonary infection, prolonged chest drainage, impaired liver function, and prolonged postoperative air leak due to pleural adhesions and pulmonary bullae. For these patients, it is difficult to shorten the PLOS using ERAS. These patients should not be included in the standard ERAS protocol; however, their PLOS can be shortened using improved techniques and methodology.

We compared various factors in the perioperative period of the two subgroups (PLOS ≤10 days and PLOS >10 days), and factors with significant differences in univariable analysis were included in a multivariable logistic regression model. We found that a surgical duration >240 min, age >64 years, surgical complication grade >2, and severe comorbidities were risk factors for increased PLOS. This result is consistent with previous studies (5, 22–25). Inclusion of these risk factors in the regression model would enable better prediction of delayed patient discharges and facilitate the development of personalized ERAS protocols by physicians and their patients. Patients with a high positive predictive value should be more carefully evaluated, and observed for longer periods of time, for each important ERAS measure to improve quality control rather than simply including them in the standard ERAS protocol.

Some limitations of our study should be considered while interpreting the results. This was a retrospective single-center study, and the conclusion was based on the single-center ERAS mode. Larger multicentre studies are required to investigate the applicability of our findings to other ERAS pathways.

## Conclusions

The optimal planned discharge time for the ERAS pathway for esophagectomy should be between 7 and 10 days postoperatively, with a 4-day discharge observation window. For patients with risk factors for delayed discharge, prediction methods should be implemented to determine the planned PLOS. Individualized perioperative management protocols should also be provided.

## Data Availability

The raw data supporting the conclusions of this article will be made available by the authors, without undue reservation.
